# Exploring Alice in Wonderland syndrome in adults with persistent headache after COVID-19: a cross-sectional study in Latin America

**DOI:** 10.1186/s12883-025-04422-y

**Published:** 2025-10-02

**Authors:** Fhabián S. Carrión-Nessi, Luis C. Ascanio, Óscar D. Omaña-Ávila, Oriana A. Regalado-Gutiérrez, Daniela L. Mendoza-Millán, Natasha A. Camejo-Ávila, David A. Forero-Peña, Alberto E. Paniz-Mondolfi

**Affiliations:** 1 Biomedical Research and Therapeutic Vaccines Institute, Ciudad Bolívar, Venezuela; 2 Department of Infectious Diseases and Tropical Medicine, Incubadora Venezolana de la Ciencia, Barquisimeto, Venezuela; 3 “Luis Razetti” School of Medicine, Universidad Central de Venezuela, Caracas, Venezuela; 4 Department of Pathology, Molecular and Cell-Based Medicine, Icahn School of Medicine at Mount Sinai, New York City, NY USA; 5 Department of Infectious Diseases, Hospital Universitario de Caracas, Caracas, Venezuela

**Keywords:** Alice in Wonderland Syndrome, Primary Headache Disorders, SARS-CoV-2, COVID-19, Post-Acute COVID-19 Syndrome, Cross-Sectional Survey, Latin America

## Abstract

**Background:**

Alice in Wonderland syndrome (AIWS) is a neuropsychiatric disorder characterized by sensory perception distortions, including altered body image perception and distortions of shape, size, motion, color, and speed. Migraine and infectious diseases are among the most common etiologies of AIWS. However, it has not been studied in individuals with persistent headache after COVID-19.

**Methods:**

This cross-sectional study included a subset of individuals with AIWS symptoms derived from a survey conducted in Latin America to identify adults with persistent headache after COVID-19. For data analysis, AIWS individuals were characterized by sex and analyzed using univariable tests. Subsequently, the entire study cohort was stratified into two groups: the AIWS group and the non-AIWS group. Binomial logistic regression using the backward stepwise selection method was performed to identify the factors associated with AIWS after COVID-19.

**Results:**

Out of 421 participants with persistent headache after COVID-19, 106 (25.2%) reported at least one AIWS symptom. The AIWS group was significantly younger (median age 36 vs. 39 years, *p* = 0.011) and had a higher proportion of pre-existing migraine (40.6% vs. 29.5%, *p* = 0.035) compared to the non-AIWS group. The most common post-COVID-19 AIWS symptoms were time distortion (32.1%), derealization/depersonalization (24.5%), and hyperchromatopsia (20.8%). Logistic regression analysis revealed that experiencing any AIWS symptom during acute COVID-19 was the strongest predictor for post-acute AIWS (OR = 9.937, 95% CI = 5.603–17.62, *p* <0.001). Other significant predictors included phonophobia (OR = 2.322, 95% CI = 1.288–4.185, *p* = 0.005) and depressive symptoms (OR = 1.937, 95% CI = 1.099–3.413, *p* = 0.022) during acute COVID-19.

**Conclusion:**

In this cohort, AIWS was a notable feature in adults with persistent headache after COVID-19, particularly in younger individuals with a history of migraine. Experiencing AIWS symptoms during acute infection increased the odds of post-acute AIWS symptoms nearly tenfold, suggesting SARS-CoV-2 may be a potent trigger. Clinicians should be aware of this association and screen for perceptual disturbances in patients with post-COVID-19 neurological sequelae.

**Supplementary Information:**

The online version contains supplementary material available at 10.1186/s12883-025-04422-y.

## Background

Alice in Wonderland syndrome (AIWS) is a neuropsychiatric disorder characterized by sensory perception distortions [1]. In 1952, neurologist Caro W. Lippman described transient episodes of altered body image perception and perceptual distortions of shape, size, motion, color, and speed in seven migraine patients [2]. British psychiatrist John Todd first coined the term AIWS in 1955 to describe these infrequent neuropsychiatric symptoms, drawing parallels with the protagonist’s experiences in Lewis Carroll’s “Alice’s Adventures in Wonderland” [3]. This syndrome seems to be more prevalent among children and young adults [4–6]. Over 60 symptoms have been associated with AIWS, including 42 visual and 16 somesthetic symptoms, among others. However, patients typically experience distorted perceptions of shape (metamorphopsia), size (macropsia or micropsia), distance (pelopsia or teleopsia), motion (kinetopsia), or color (achromatopsia) [7].

AIWS has been associated with a variety of conditions, including infectious diseases (Epstein-Barr virus, cytomegalovirus), central nervous system lesions (tumor or vascular), psychiatric disorders (major depressive disorder, schizophrenia), paroxysmal neurological disorders (migraine, epilepsy), certain medications (risperidone, oseltamivir), and recreational or abused drugs (cannabis, cocaine), among others [7]. However, these etiologies have only been described in case reports and case series. Recent studies suggest that infectious etiology, particularly viral infections, is the primary cause of AIWS, especially in children [6–8], and symptoms may manifest before, during, or after the primary infection is recognized.

As of September 2, 2025, the coronavirus disease 2019 (COVID-19) had affected over 778 million individuals and resulted in more than 7 million deaths globally [9]. It is estimated that over 10% of patients persist with COVID-19 symptoms, such as fatigue, dyspnea, myalgia, muscle weakness, and/or depression, even months after infection [10]. Neurological symptoms, such as cognitive disturbances and persistent headache are common during the acute phase of the disease [11, 12], and may persist for more than six months [13, 14]. Persistent headache prevalence ranges from 8 to 15% within six months after COVID-19 [15]. However, our understanding of the clinical spectrum and predisposing factors of persistent headache after severe acute respiratory syndrome coronavirus 2 (SARS-CoV-2) infection is limited [16–19]. Despite the association of AIWS with neurological and infectious etiologies, the characteristics of these sensory perception distortions have not been thoroughly studied in patients with COVID-19.

Information on the association between AIWS and SARS-CoV-2 infection is limited to a few case reports. One report documented a 5-year-old boy who developed seizures manifesting as teleopsia two weeks after a SARS-CoV-2 infection, though a causal link cannot be definitively established from a single case [20]. Another case series described three children with self-limited visual disturbances (macropsia, micropsia, teleopsia, and pelopsia) in the post-infectious period, further suggesting a possible association [21]. The limited evidence available and the potential relationship between AIWS and SARS-CoV-2 infection encouraged us to conduct this research. Therefore, this study aims to characterize the clinical features of AIWS and identify its associated factors in a large cohort of individuals with persistent headache after SARS-CoV-2 infection.

## Methods

### Study design

This cross-sectional study used a subset of data derived from a survey conducted in Venezuela and other Latin American countries between April 15^th^ and 30^th^, 2022. The survey, hosted on the “Google Forms” platform (Google LLC, Mountain View, CA, USA), aimed to identify adults who experienced persistent headache after SARS-CoV-2 infection [19]. Eligible participants were individuals aged 18 years and older who tested positive for SARS-CoV-2 infection via reverse transcriptase polymerase chain reaction or antigen tests [22] and had persistent headache in the post-acute phase of the infection. Following established definitions, “acute COVID-19” was defined as the first four weeks from symptom onset [23]. “Persistent headache” was defined as headache lasting for more than 28 days following the initial infection, meaning all participants were assessed regarding symptoms present in the post-acute phase. To further characterize this timing, the median duration from the onset of persistent headache to survey completion was 159 (IQR: 88–347) days. The study population comprised a subset of participants who met the inclusion criteria and reported experiencing at least one AIWS symptom after SARS-CoV-2 infection. As there are no established diagnostic criteria for AIWS, symptoms were defined based on descriptions in published studies [8, 24, 25]. Participants were excluded if their responses to certain conditional items were inconsistent or incomplete [19].

The survey link was disseminated through a non-probabilistic, convenience sampling approach. It was shared via email and social media platforms (Facebook, Instagram, Twitter) by several medical societies, patient advocacy groups, and academic institutions across Latin America. The link was also cascaded through the instant messaging application WhatsApp among professional and patient networks. To encourage broad participation, the survey was anonymous and voluntary.

### Survey design and data collection

The survey, conducted in Spanish and previously published in another paper, consisted of 37 questions divided into four sections: demographics, past medical history, clinical characteristics of persistent headache, and COVID-19 vaccination status [19]. The primary focus of the survey was on the clinical characteristics of persistent headache. However, it also included questions about AIWS concomitant symptoms during acute COVID-19 (item 10) and after COVID-19 (item 33) [19]. The AIWS symptoms are summarized in Table 1.

**Table 1:** Summary of AIWS symptoms

Symptom	Description
Macrosomatognosia or microsomatognosia	Sensation of the entire body or a body part being larger (macrosomatognosia) or smaller (microsomatognosia) than its actual size
Macropsia or micropsia	Perception of objects as larger as (macropsia) or smaller than (micropsia) their actual size
Teleopsia or pelopsia	Perception of objects as being further away (teleopsia) or closer than (pelopsia) they actually are
Derealization or depersonalization	Sensation of the world (derealization) or oneself (depersonalization) being unreal
Achromatopsia or hypochromatopsia	Total (achromatopsia) or partial (hypochromatopsia) inability to perceive colors
Time distortion	Sensation of time speeding up or slowing down
Dysmorphopsia	Perception of lines and contours as wavy
Hyperchromatopsia	Perception of colors as overly bright
Illusory levitation	Sensation of floating in the air
Dyschromatopsia	Confusion of colors
Erythropsia	Perception of objects as red

The questions were designed as closed-ended (yes/no) to facilitate understanding by the participants. Demographics included age, sex, education level, marital status, race, occupation, and country of residence. Past medical history data included COVID-19 clinical presentation, vaccination status before symptom onset, comorbidities, smoking habits, history of headache before COVID-19, and neurological and AIWS symptoms during acute COVID-19. The development of the survey, including a pilot test to ensure clarity and content relevance for the target population, is described in a previous study [19].

### Statistical analysis

Continuous data were presented as mean and standard deviation (SD) for normally distributed data or as median and interquartile range (IQR) for non-normally distributed data. The distribution of continuous variables was assessed for normality using the Kolmogorov-Smirnov test. Categorical data were presented as frequency and percentage (%).

For the primary analysis, the study cohort was stratified into two groups: an “AIWS group” (participants reporting at least one AIWS symptom after COVID-19) and a “non-AIWS group”. Univariable analyses were performed to compare these groups using the Mann-Whitney U test for non-normally distributed continuous variables, Student’s *t*-test for normally distributed continuous variables, and Pearson’s chi-squared or Fisher’s exact test for categorical variables. A two-sided *p* value less than 0.05 was considered statistically significant.

To identify factors associated with the presence of AIWS after COVID-19, multivariable binomial logistic regression was performed. Variables that were statistically significant (*p* <0.05) in the univariable analyses were entered as candidate predictors into the model. A backward stepwise (Wald) selection method was used to derive the final model. Model fit was assessed using the Hosmer-Lemeshow test, and the explanatory power was evaluated with the Nagelkerke R² statistic. The survey instrument was designed to require a response for all items included as candidate predictors in the regression models, thus preventing missing data on this subset of variables. Consequently, all 421 participants were included in the final analyses, and data imputation was not necessary. Statistical analyses were performed using SPSS version 26 (IBM Corporation, Armonk, NY, USA). Figures were generated using GraphPad Prism version 10.1.2 (GraphPad Software, Boston, MA, USA).

## Results

**Fig. 1:**
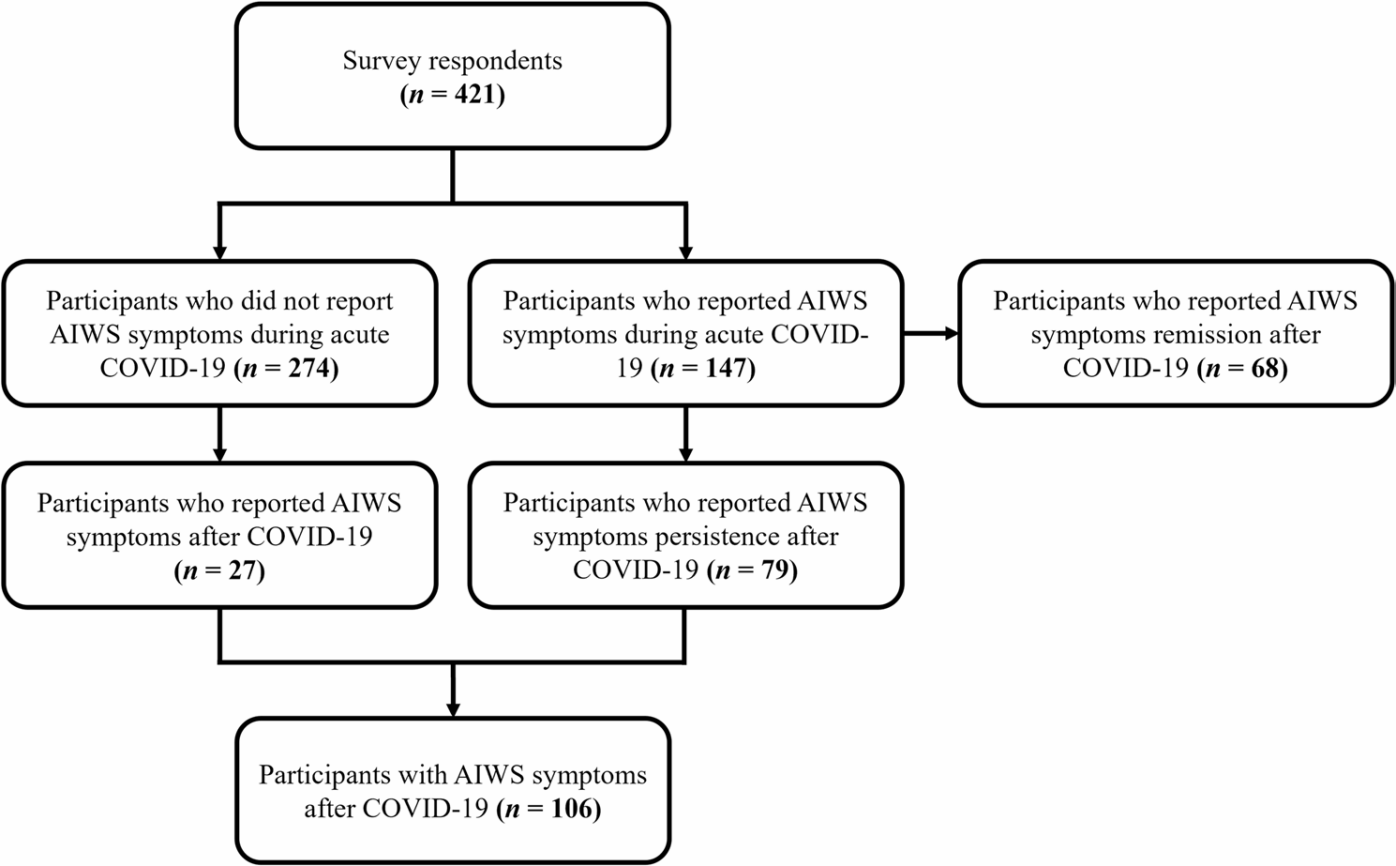
Flowchart of the participants’ selection. *AIWS* Alice in Wonderland syndrome, *COVID-19* coronavirus disease 2019

Out of the 421 survey respondents, 147 (34.9%) reported AIWS symptoms during acute COVID-19. Out of these, 79/147 (53.7%) participants reported persistence of AIWS symptoms into the post-acute phase (defined as > four weeks post-infection), while 68/147 (46.3%) participants reported AIWS symptoms remission after COVID-19. Additionally, 274 (65.1%) participants did not report AIWS symptoms during acute COVID-19. However, 27/274 (9.9%) of these participants reported AIWS symptoms after COVID-19 (Fig. 1).

### Participants’ demographics

A total of 106 participants (Fig. 1) from eight Latin American countries informed experiencing at least one AIWS symptom along with persistent headache after COVID-19. The median age was 36 (IQR: 16, range: 18–65) years, mostly female (84%, *n* = 89), with a college education degree (70.7%, *n* = 75), and self-identifying as mixed race (57.5%, *n* = 61). The demographics were not statistically different between male and female participants (Table 2).

**Table 2:** Demographics of 106 participants with at least one AIWS symptom after SARS-CoV-2 infection by sex

Demographics	All (*n* = 106, 100%)	Female (*n* = 89, 84%)	Male (*n* = 17, 16%)	*p* value
Age, median (IQR), years	36 (16)	36 (15)	42 (17)	0.133^a^
Education level, *n* (%)				0.703^b^
High school	16 (15.1)	13 (14.6)	3 (17.6)	
Associated degree	15 (14.2)	14 (15.7)	1 (5.9)	
University	75 (70.7)	62 (69.7)	13 (76.5)	
Marital status, *n* (%)				0.515^b^
Single	47 (44.4)	41 (46.1)	6 (35.3)	
Married	42 (39.6)	35 (39.3)	7 (41.2)	
Common-law marriage	14 (13.2)	11 (12.4)	3 (17.6)	
Divorced	3 (2.8)	2 (2.2)	1 (5.9)	
Race, *n* (%)				0.055^b^
Mixed	61 (57.5)	47 (52.8)	14 (82.4)	
White	44 (41.5)	41 (46.1)	3 (17.6)	
Black	1 (1)	1 (1.1)	0 (0)	
Occupation, *n* (%)				0.754^b^
Healthcare worker	32 (30.1)	27 (30.3)	5 (29.4)	
Employee	29 (27.4)	23 (25.8)	6 (35.3)	
Self-employed	25 (23.6)	20 (22.5)	5 (29.4)	
Student	15 (14.2)	14 (15.7)	1 (5.9)	
Unemployed/Retired	5 (4.7)	5 (5.6)	0 (0)	
Country of residence, *n* (%)				0.186^b^
Argentina	2 (1.9)	2 (2.2)	0 (0)	
Chile	1 (0.9)	1 (1.1)	0 (0)	
Colombia	15 (14.2)	12 (13.5)	3 (17.6)	
Ecuador	1 (0.9)	1 (1.1)	0 (0)	
Mexico	9 (8.5)	6 (6.7)	3 (17.6)	
Peru	3 (2.8)	1 (1.1)	2 (11.8)	
Uruguay	1 (0.9)	1 (1.1)	0 (0)	
Venezuela	74 (69.9)	65 (73)	9 (52.9)	

^a^Mann-Whitney U test

^b^Fisher’s exact test

### Past medical history and AIWS symptoms during acute COVID-19

Over 90% (*n* = 98) of participants reported having had mild to moderate COVID-19. Most participants were vaccinated against COVID-19 before symptoms’ onset (56.6%, *n* = 60). Hypertension (20.8%, *n* = 22) was the most common comorbidity, followed by asthma (8.5%, *n* = 9), diabetes (4.7%, *n* = 5), and hypothyroidism (2.8%, *n* = 3). Male participants had a significantly higher history of smoking compared to female participants (17.6% vs. 4.5%, *p* = 0.045). The mean pack-year index among smoking participants was 2.4 (SD: 2.9). Female participants had a significantly higher proportion of headache history, primarily of the migraine type (40.6%), before COVID-19 in comparison to male participants (65.2% vs. 35.3%, *p* = 0.021) (Table 3).

**Table 3:** Past medical history of 106 participants with at least one AIWS symptom after SARS-CoV-2 infection by sex

Past medical history	All (*n* = 106, 100%)	Female (*n* = 89, 84%)	Male (*n* = 17, 16%)	*p* value
Clinical presentation of COVID-19, *n* (%)				0.777^a^
Mild/Moderate	98 (92.5)	82 (92.1)	16 (94.1)	
Severe/Critical	8 (7.5)	7 (7.9)	1 (5.9)	
Vaccination status before symptom’s onset, *n* (%)				0.161^a^
No	46 (43.4)	36 (40.4)	10 (58.8)	
Yes	60 (56.6)	53 (59.6)	7 (41.2)	
Comorbidities, yes (%)				
Hypertension	22 (20.8)	18 (20.2)	4 (23.5)	0.758^a^
Asthma	9 (8.5)	8 (9)	1 (5.9)	0.674^a^
Diabetes	5 (4.7)	3 (3.4)	2 (11.8)	0.181^b^
Hypothyroidism	3 (2.8)	3 (3.4)	0 (0)	1^b^
HIV	1 (0.9)	0 (0)	1 (5.9)	0.16^b^
Cancer	1 (0.9)	1 (1.1)	0 (0)	1^b^
COPD	1 (0.9)	1 (1.1)	0 (0)	1^b^
CKD	1 (0.9)	1 (1.1)	0 (0)	1^b^
Smoking, *n* (%)				0.045^a^
No	99 (93.4)	85 (95.5)	14 (82.4)	
Yes	7 (6.6)	4 (4.5)	3 (17.6)	
Pack-year index, mean (SD)	2.4 (2.9)	2.1 (2.3)	2.7 (4.2)	0.808^c^
History of headache before COVID-19, *n* (%)				0.021^a^
No	42 (39.6)	31 (34.8)	11 (64.7)	
Yes	64 (60.4)	58 (65.2)	6 (35.3)	
Migraine, yes (%)	43 (40.6)	39 (43.8)	4 (23.5)	0.118^a^
Tension, yes (%)	24 (22.6)	22 (24.7)	2 (11.8)	0.242^a^
Other, yes (%)	4 (3.8)	4 (4.5)	0 (0)	1^b^
Concomitant AIWS symptoms during acute COVID-19, yes (%)				
Macrosomatognosia or microsomatognosia total or partial body	17 (16)	14 (15.7)	3 (17.6)	0.844^a^
Macropsia or micropsia	6 (5.7)	4 (4.5)	2 (11.8)	0235^a^
Teleopsia or pelopsia	17 (16)	12 (13.5)	5 (29.4)	0.101^a^
Derealization or depersonalization	28 (26.4)	25 (28.1)	3 (17.6)	0.371^a^
Achromatopsia or hypochromatopsia	4 (3.8)	4 (4.5)	0 (0)	1^b^
Time distortion	32 (30.2)	29 (32.6)	3 (17.6)	0.219^a^
Dysmorphopsia	8 (7.5)	6 (6.7)	2 (11.8)	0.472^a^
Hyperchromatopsia	13 (12.3)	13 (14.6)	0 (0)	0.093^a^
Illusory levitation	22 (20.8)	21 (23.6)	1 (5.9)	0.099^a^
Dyschromatopsia	2 (1.9)	2 (2.2)	0 (0)	1^b^
Erythropsia	4 (3.8)	3 (3.4)	1 (5.9)	0.508^b^
Other concomitant symptoms during acute COVID-19, *n* (%)				
Depressive symptoms	57 (53.8)	52 (58.4)	5 (29.4)	0.028^a^
Phonophobia	61 (57.5)	54 (60.7)	7 (41.2)	0.136^a^
Osmophobia	28 (26.4)	23 (25.8)	5 (29.4)	0.76^a^
Paresthesia	57 (53.8)	52 (58.4)	5 (29.4)	0.028^a^
Anxious symptoms	69 (65.1)	62 (69.7)	7 (41.2)	0.024^a^
Palpebral edema	11 (10.4)	11 (12.4)	0 (0)	0.126^a^
Mental fog	63 (59.4)	57 (64)	6 (35.3)	0.027^a^
Photophobia	56 (52.8)	52 (58.4)	4 (23.5)	0.008^a^
Fainting	2 (1.9)	2 (2.2)	0 (0)	1^b^
Upper eyelid drooping and/or pupillary constriction	4 (3.8)	4 (4.5)	0 (0)	1^b^
Ageusia or hypogeusia	43 (40.6)	35 (39.3)	8 (47.1)	0.552^a^
Anosmia or hyposmia	42 (39.6)	34 (38.2)	8 (47.1)	0.494^a^
Tinnitus	45 (42.5)	40 (44.9)	5 (29.4)	0.235^a^
Dysgeusia	30 (28.3)	26 (29.2)	4 (23.5)	0.634^a^
Fatigue	91 (85.8)	76 (85.4)	15 (88.2)	0.758^a^
Face or forehead sweating	28 (26.4)	25 (28.1)	3 (17.6)	0.371^a^
Myalgia	74 (69.8)	65 (73)	9 (52.9)	0.098^a^
Sleep problems	63 (59.4)	55 (61.8)	8 (47.1)	0.257^a^
Nasal congestion	46 (43.4)	40 (44.9)	6 (35.3)	0.462^a^
Tearing	22 (20.8)	20 (22.5)	2 (11.8)	0.319^a^
Nausea	43 (40.6)	41 (46.1)	2 (11.8)	0.008^a^
Vomiting	16 (15.1)	15 (16.9)	1 (5.9)	0.247^a^
Dizziness	32 (30.2)	32 (36)	0 (0)	0.003^a^
Fever	43 (40.6)	37 (41.6)	6 (35.3)	0.629^a^

*HIV* human immunodeficiency virus, *COPD* chronic obstructive pulmonary disease, *CKD* chronic kidney disease

^a^Pearson’s chi-square test

^b^Fisher’s exact test

^c^Student’s *t* test for independent samples

The median number of AIWS symptoms during acute COVID-19 for the 79/106 (74.5%) participants was 2 (IQR: 1, range: 1–7). The most frequent AIWS symptoms were time distortion (30.2%, *n* = 32), derealization or depersonalization (26.4%, *n* = 28), illusory levitation (20.8%, *n* = 22), macrosomatognosia or microsomatognosia (16%, *n* = 17), and teleopsia or pelopsia (16%, *n* = 17). The least frequent were dyschromatopsia (1.9%, *n* = 2), erythropsia (3.8%, *n* = 4), and achromatopsia or hypochromatopsia (3.8%, *n* = 4). There were no statistically significant differences between the AIWS symptoms during acute COVID-19 of male and female participants (Table 3).

The most common concomitant symptoms during acute COVID-19 were fatigue (85.8%, *n* = 91), myalgia (69.8%, *n* = 74), anxiety-related symptoms (65.1%, *n* = 69), sleep problems (59.4%, *n* = 63), and mental fog (59.4%, *n* = 63). Female participants had a significantly higher proportion of depression-associated symptoms (58.4% vs. 29.4% *p* = 0.028), paresthesia (58.4% vs. 29.4%, *p* = 0.028), anxiety-related symptoms (69.7% vs. 41.2%, *p* = 0.024), mental fog (64% vs. 35.3%, *p* = 0.027), photophobia (58.4% vs. 23.5%, *p* = 0.008), nausea (46.1% vs. 11.8%, *p* = 0.008), and dizziness (36% vs. 0%, *p* = 0.003) compared to male participants (Table 3).

### AIWS symptoms after COVID-19

**Fig. 2:**
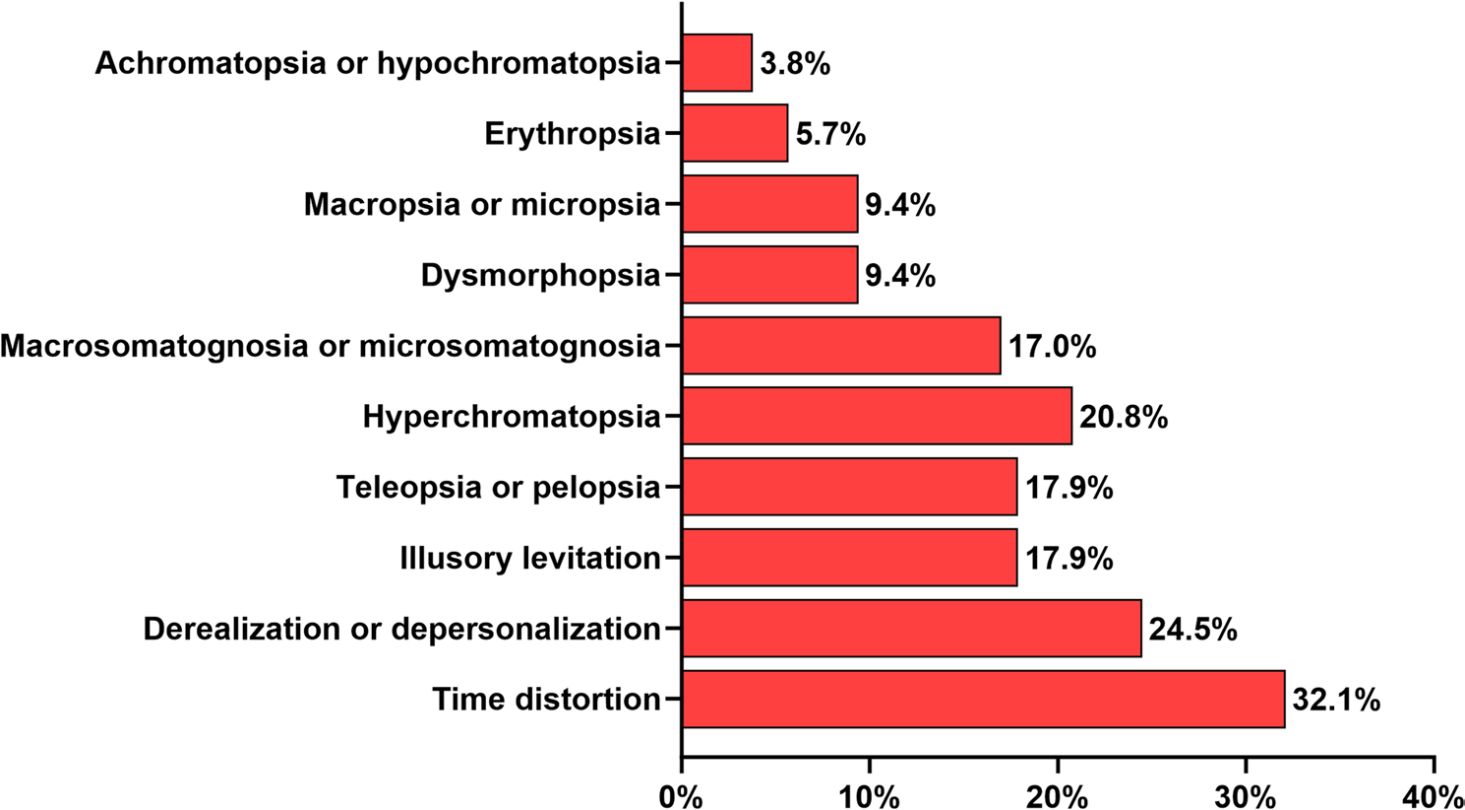
Frequency of AIWS symptoms after COVID-19

The median number of AIWS symptoms after COVID-19 among 106 participants was 1 (IQR: 1, range: 1–7). Time distortion (32.1%, *n* = 34), derealization or depersonalization (24.5%, *n* = 26), hyperchromatopsia (20.8%, *n* = 22), illusory levitation (17.9%, *n* = 19), and teleopsia or pelopsia (17.9%, *n* = 19) were the most frequent symptoms. The least frequent were achromatopsia or hypochromatopsia (3.8%, *n* = 4), erythropsia (5.7%, *n* = 6), and macropsia or micropsia (9.4%, *n* = 10) (Fig. 2). There were no statistically significant differences between the AIWS symptoms after COVID-19 of male and female participants (Supplementary Data 1).

Out of the 79 participants who reported AIWS symptoms persistence after COVID-19, 41 (51.9%) reported no change in the number of AIWS symptoms. Among these 41/79 participants, 30/79 (73.2%) reported having the same symptoms experienced during acute COVID-19 and the remaining 11/79 (26.8%) reported having different symptoms from those experienced during acute COVID-19. Conversely, 27/79 (34.2%) reported a decrease in the number of AIWS symptoms and 11/79 (13.9%) experienced an increase in the number of AIWS symptoms.

### Comparison between AIWS and non-AIWS groups

There were 106 (25.2%) participants who reported experiencing at least one AIWS symptom along with persistent headache after SARS-CoV-2 infection and 315 (74.8%) participants who reported no AIWS symptoms after COVID-19. Participants in the AIWS group were significantly younger, with a median age of 36 (IQR: 16) years (*p* = 0.011). The remaining demographics did not significantly differ between groups (Supplementary Data 2).

Regarding past medical history, there were no statistically significant differences between groups regarding COVID-19 severity (*p* = 0.418), COVID-19 vaccination status (*p* = 0.364), comorbidities, including hypertension (*p* = 0.803), asthma (*p* = 0.939), diabetes (*p* = 0.356), and hypothyroidism (*p* = 0.859), or smoking (*p* = 0.894). A key difference emerged in pre-existing headache diagnoses: participants in the AIWS group had a significantly higher proportion of pre-COVID-19 migraine compared to the non-AIWS group (40.6% vs. 29.5%, *p* = 0.035) (Table 4). There was a moderately positive correlation (ρ = 0.455) by Spearman’s rank correlation between the number of AIWS symptoms during acute COVID-19 and after COVID-19 (*p* <0.001).

**Table 4:** Past medical history between AIWS and non-AIWS groups during SARS-CoV-2 infection

Past medical history	AIWS (*n* = 106, 25.2%)	Non-AIWS (*n* = 315, 74.8%)	*p* value
Clinical presentation of COVID-19, *n* (%)			0.418^a^
Mild/Moderate	98 (92.5)	298 (94.6)	
Severe/Critical	8 (7.5)	17 (5.4)	
COVID-19 vaccination status before symptom’s onset, *n* (%)			0.364^a^
No	46 (43.4)	121 (38.4)	
Yes	60 (56.6)	194 (61.6)	
Comorbidities, yes (%)			
Hypertension	22 (20.8)	69 (21.9)	0.803^a^
Asthma	9 (8.5)	26 (8.3)	0.939^a^
Diabetes	5 (4.7)	9 (2.9)	0.356^a^
Hypothyroidism	3 (2.8)	10 (3.2)	0.859^a^
HIV	1 (0.9)	3 (1)	1^b^
Cancer	1 (0.9)	3 (1)	1^b^
COPD	1 (0.9)	1 (0.9)	1^b^
CKD	1 (0.9)	0 (0)	0.252^b^
Smoking, *n* (%)			0.894^a^
No	99 (93.4)	293 (93)	
Yes	7 (6.6)	22 (7)	
Pack-year index, mean (SD)	2.4 (2.9)	4.9 (6.5)	0.32^c^
History of headache before COVID-19, *n* (%)			0.639^a^
No	42 (39.6)	133 (42.2)	
Yes	64 (60.4)	182 (57.8)	
Migraine, yes (%)	43 (40.6)	93 (29.5)	0.035^a^
Tension, yes (%)	24 (22.6)	76 (24.1)	0.756^a^
Other, yes (%)	4 (3.8)	25 (7.9)	0.143^a^
Other concomitant symptoms during acute COVID-19, yes (%)			
Depressive symptoms	57 (53.8)	87 (27.6)	<0.001^a^
Phonophobia	61 (57.5)	90 (28.6)	<0.001^a^
Osmophobia	28 (26.4)	45 (14.3)	0.004^a^
Paresthesia	57 (53.8)	110 (34.9)	0.001^a^
Anxious symptoms	69 (65.1)	138 (43.8)	<0.001^a^
Palpebral edema	11 (10.4)	12 (3.8)	0.01^a^
Mental fog	63 (59.4)	135 (42.9)	0.003^a^
Photophobia	56 (52.8)	110 (34.9)	0.001^a^
Fainting	2 (1.9)	4 (1.3)	0.644^a^
Upper eyelid drooping and/or pupillary constriction	4 (3.8)	7 (2.2)	0.386^a^
Ageusia or hypogeusia	43 (40.6)	104 (33)	0.158^a^
Anosmia or hyposmia	42 (39.6)	112 (35.6)	0.452^a^
Tinnitus	45 (42.5)	97 (30.8)	0.028^a^
Dysgeusia	30 (28.3)	53 (16.8)	0.01^a^
Fatigue	91 (85.8)	253 (80.3)	0.203^a^
Face or forehead sweating	28 (26.4)	79 (25.1)	0.785^a^
Myalgia	74 (69.8)	189 (60)	0.071^a^
Sleep problems	63 (59.4)	163 (51.7)	0.17^a^
Nasal congestion	46 (43.4)	152 (48.3)	0.386^a^
Tearing	22 (20.8)	43 (13.7)	0.08^a^
Nausea	43 (40.6)	68 (21.6)	<0.001^a^
Vomiting	16 (15.1)	26 (8.3)	0.042^a^
Dizziness	32 (30.2)	63 (20)	0.03^a^
Fever	43 (40.6)	111 (35.2)	0.325^a^

^a^Pearson’s chi-square test

^b^Fisher’s exact test

^c^Student’s *t* test for independent samples

During acute COVID-19, participants in the AIWS group had a significantly higher proportion of depressive symptoms (53.8% vs. 27.6%, *p* <0.001), phonophobia (57.5% vs. 28.6%, *p* <0.001), osmophobia (26.4% vs. 14.3%, *p* = 0.004), paresthesia (53.8% vs. 34.9%, *p* = 0.001), anxious symptoms (65.1% vs. 43.8%, *p* <0.001), palpebral edema (10.4% vs. 3.8%, *p* = 0.01), mental fog (59.4% vs. 42.9%, *p* = 0.003), photophobia (52.8% vs. 34.9%, *p* = 0.001), tinnitus (42.5% vs. 30.8%, *p* = 0.028), dysgeusia (28.3% vs. 16.8%, *p* = 0.01), nausea (40.6% vs. 21.6%, *p* <0.001), vomiting (15.1% vs. 8.3%, *p* = 0.042), and dizziness (30.2% vs. 20%, *p* = 0.03) compared to non-AIWS group (Table 4).

### Concomitant symptoms associated with AIWS during acute COVID-19 and after COVID-19

**Fig. 3:**
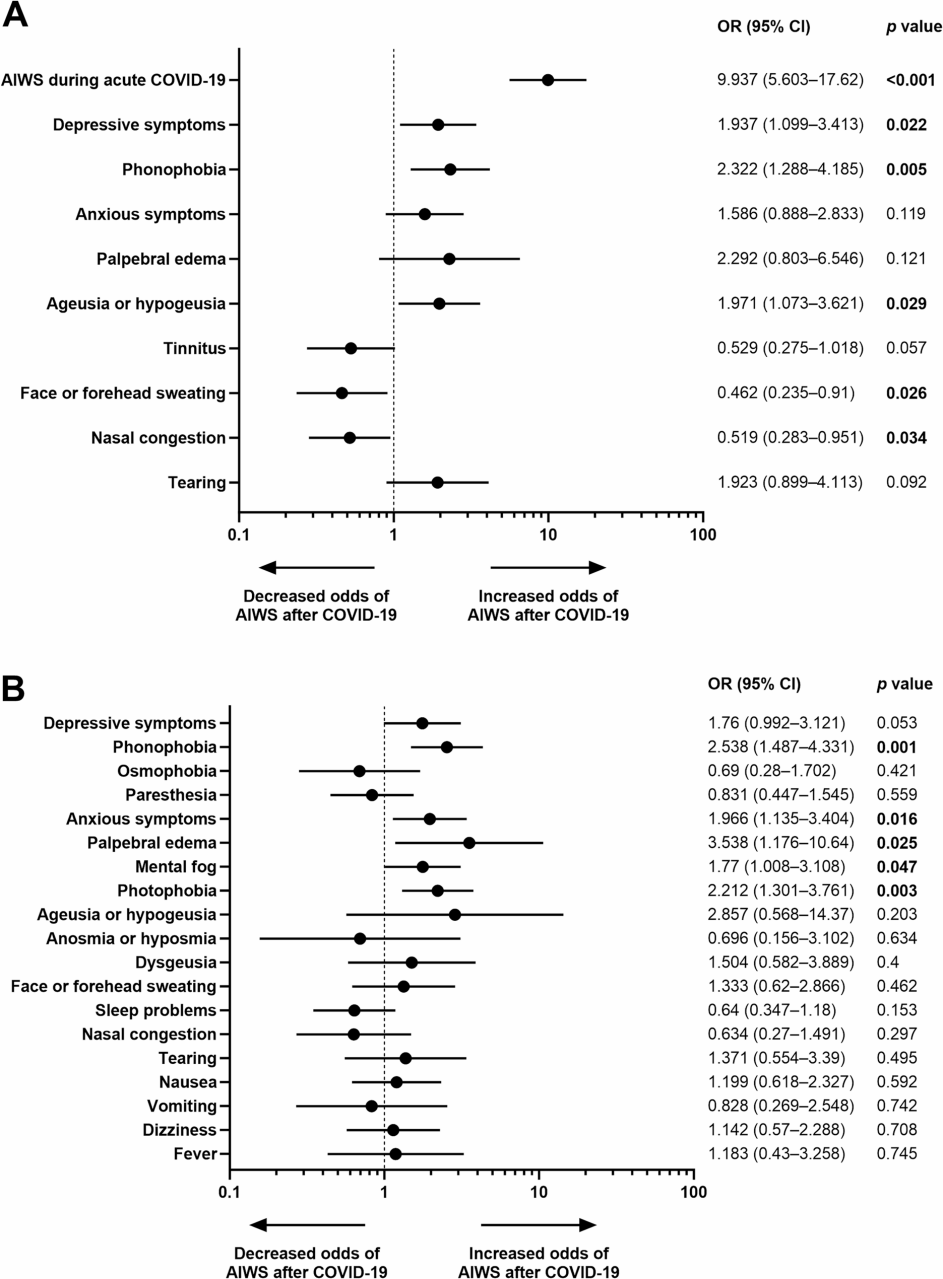
Factors associated with AIWS after COVID-19. **A** Association between symptoms during acute COVID-19 and the odds of post-COVID-19 AIWS. **B** Association between concurrent post-COVID-19 symptoms and the odds of post-COVID-19 AIWS. Odds ratios (OR) > 1 indicate increased odds that a patient experienced post-COVID AIWS, while odds ratios < 1 indicate decreased odds. The black circles represent the adjusted OR for each factor, and the lines represent the 95% confidence intervals. For all symptoms listed, the reference category was the absence of that symptom. Variables with statistically significant associations (*p* <0.05) are indicated in bold

Regarding concomitant symptoms during acute COVID-19, the best valid model (*p* <0.001, R^2^ Nagelkerke = 0.391, Hosmer-Lemeshow test = 0.342), which correctly classified 82.4% of the participants (347 out of 421), found that having at least one AIWS symptom during acute COVID-19 (OR = 9.937, 95% CI = 5.603–17.62, *p* <0.001), depressive symptoms (OR = 1.937, 95% CI = 1.099–3.413, *p* = 0.022), phonophobia (OR = 2.322, 95% CI = 1.288–4.185, *p* = 0.005), and ageusia or hypogeusia (OR = 1.971, 95% CI = 1.073–3.621, *p* = 0.029) increased odds of having AIWS symptoms after COVID-19, whereas face or forehead sweating (OR = 0.462, 95% CI = 0.235–0.91. *p* = 0.026) and nasal congestion (OR = 0.519, 95% CI = 0.283–0.951, *p* = 0.034) decreased odds of having AIWS symptoms after COVID-19 (Fig. 3A).

Regarding concomitant symptoms after COVID-19, the best valid model (*p* <0.001, R^2^ Nagelkerke = 0.263, Hosmer-Lemeshow test = 0.688), which classified 79.3% of the participants (334 out of 421), found that having phonophobia (OR = 2.538, 95% CI = 1.487–4.331, *p* = 0.001), anxious symptoms (OR = 1.966, 95% CI = 1.135–3.404, *p* = 0.016), palpebral edema (OR = 3.538, 95% CI = 1.176–10.64, *p* = 0.025), mental fog (OR = 1.77, 95% CI = 1.008–3.108, *p* = 0.047), and photophobia (OR = 2.212, 95% CI = 1.301–3.761, *p* = 0.003) increased odds of having AIWS symptoms after COVID-19 (Fig. 3B).

To assess the potential influence of selection bias from the large proportion of healthcare workers in our sample (30.1%), we conducted a sensitivity analysis by repeating the logistic regression on the imputed datasets with this subgroup excluded. This analysis shows that the primary predictors of post-COVID-19 AIWS symptoms remained largely consistent in direction and statistical significance (Supplementary Data 3).

## Discussion

This study characterized the clinical profile of 106 individuals who reported experiencing at least one AIWS symptom along with persistent headache after SARS-CoV-2 infection in Latin America. To the best of our knowledge, there is limited data on the association of AIWS and COVID-19 except for some case reports [21, 26, 27]. A majority of individuals who reported AIWS symptoms during acute COVID-19 continued to experience these symptoms in the post-acute phase. While our survey did not capture the frequency (i.e., whether persistent or intermittent/paroxysmal), this finding highlights a chronicity to the phenomenon that extends beyond the initial infection. Moreover, some individuals who did not exhibit any AIWS symptoms during acute COVID-19, later experienced at least one symptom after the acute phase resolved. This highlights the importance of considering SARS-CoV-2 as a potential trigger for AIWS.

Among adults, migraines have been the most prevalent condition associated with AIWS, followed by infectious etiologies [7, 28]. Studies on the association of headache with AIWS have estimated a prevalence of around 15% of this syndrome among patients with migraine [28–30]. Our study found that individuals who reported AIWS symptoms had a higher proportion of migraine history in contrast to their opposite group. A possible hypothesis lies in the role of the depolarization wave that disseminates during a migraine triggering the extracellular release of potassium and calcium ions, nitric oxide, and arachidonic acid, which then activate meningeal nociceptors. These nociceptors are known to project nerve fibers to the thalamus and, consequently, to the sensory cortex potentially translating into AIWS symptoms [31]. In fact, theories suggest that migraine patients are more susceptible to dissociative symptoms [32] and AIWS due to observed cortical changes in temporoparietal regions and junctions [24], and alterations in neurotransmitters such as serotonin and glutamate [33]. These findings could inform potential therapeutic strategies for AIWS-related symptoms. Most of our participants were female, which has been identified as a risk factor for migraine [34] and, although AIWS has no sex preference [28, 35], migraine-AIWS-associated cases have shown a higher prevalence in female patients [31]. However, in our study, sex did not yield a significant difference among groups.

Infectious diseases, mainly of viral etiology, have also been associated with this syndrome, but the available information on AIWS in patients with COVID-19 is almost nil. Most of our participants with AIWS symptoms after COVID-19 had a mild to moderate form of infection. In our study, having symptoms such as ageusia/hypogeusia, phonophobia, or depressive symptoms during acute COVID-19 represented a twofold increase in the risk of developing AIWS after COVID-19. As the literature regarding the potential triggering role of COVID-19 to AIWS is scarce, no evidence has elucidated a possible explanation for this finding, and future studies would be necessary to explore a possible underlying pathophysiological mechanism. Out of 106 participants, 79 often had at least one AIWS symptom during acute COVID-19, which persisted even after disease resolution. The risk of AIWS symptoms persistence after acute COVID-19 was 10 times higher in individuals who experienced at least one AIWS symptom during acute COVID-19. This suggests that there could be a triggering relationship between the acute phase of infection and the development of AIWS. Nevertheless, 27 participants reported at least one AIWS symptom *de novo* after acute COVID-19. The most frequently observed AIWS symptoms after COVID-19 included time distortions, derealization/depersonalization, illusory levitation, and hyperchromatopsia. These symptoms align with those reported in existing literature [28, 31]. The emergence of new or persistent AIWS symptoms following initial symptomatology aligns with evidence from a case series of 48 patients [25], although lower frequencies have been observed in smaller case series [36, 37].

Our study reports a high frequency of derealization or depersonalization, confirming findings from previous studies that analyzed AIWS exclusively in migraine patients [38]. However, this surpasses the frequencies found in studies about different AIWS etiologies [7]. Recently, Fitzek et al. [39]. conducted a study on migraine patients, where 16.5% reported at least one AIWS symptom. Most patients were female, with a mean age of 42 years, and 52% experienced migraine with aura. In the same study, micropsia and/or teleopsia were the most common symptoms, contrasting with the depersonalization or derealization symptoms identified in here. However, among the core AIWS symptoms (teleopsia or micropsia, macropsia or pelopsia, microsomatognosia or macrosomatognosia) [24], teleopsia or pelopsia (17.6%) and macrosomatognosia or microsomatognosia (17.6%) were the most frequently reported symptoms in our study. Among associated symptoms, Fitzek et al. [39]. reported that the “feeling of unfamiliarity or disconnection toward their own body, sensation, or physical experiences, perceiving them as unreal and distant” was the third most common symptom. While this description may encapsulate the depersonalization or derealization reported in our study, caution should be exercised when making direct comparisons. Despite the inherent differences between the two studies, common characteristics of both study populations included a higher prevalence in female, middle-aged patients, and a history of migraine. Additionally, the subjectiveness of these symptoms (depersonalization or derealization) poses a limitation to the study due to the inability of the survey to delve further into the neurobiological context and the perceptual heterogeneity of these symptoms among patients.

The pathophysiology of AIWS remains elusive, but several mechanisms have been proposed within the context of infectious diseases as potential contributors to this syndrome. These include cerebral hypoperfusion and ischemia [40, 41], autoimmunity mediated by molecular mimicry [42], increased blood-brain barrier permeability influenced by pro-inflammatory cytokines, and direct cytopathic neuronal injury [8]. The temporo-parietal-occipital junction, implicated in the integration of visual and somatosensory stimuli, is considered the most likely brain area affected by AIWS [24]. This area is responsible for generating internal and external representations of self. Time distortion, either perceived as a slower or faster passage of time, is the most frequently reported symptom in pediatric patients [42–44] and the general population [45]. This symptom is predominantly attributed to the involvement of the right parietal and occipital areas [46–50], with some exceptions involving temporal and frontal regions [50]. Previous studies have linked each of these mechanisms to COVID-19 [51–58], suggesting that SARS-CoV-2 could potentially trigger AIWS.

In the AIWS group, the median age was lower compared to the non-AIWS group (36 vs. 39). This is consistent with descriptions of the syndrome’s typical onset during the fourth or fifth decade of life [38, 59]. Migraine is the most common cause of AIWS in adults and the second most common in children, with an estimated prevalence between 15% and 27.1% [7, 24, 25, 36, 41, 59, 60]. Our study observed a higher prevalence (40.6%) of migraine among individuals with headache history before COVID-19 within the AIWS group. Not all individuals experienced AIWS symptoms during acute COVID-19 but experiencing at least one symptom during this phase increased the odds of developing this syndrome after COVID-19 by nearly tenfold. This phenomenon could be explained by alterations in certain brain circuits in the temporo-parietal-occipital carrefour, secondary to the aforementioned pathophysiological mechanisms. The persistence of these alterations over time is attributed to a pro-inflammatory state and deregulation of the astrocyte-neuron lactate shuttle following infection [53, 54, 61].

Headaches are frequently reported as the primary cause of AIWS in adults. The majority of these instances are linked to migraines, both with and without aura, followed by tension-type headache. There is only a single report of cluster headache associated with this condition [7, 62]. In our participants cohort, the persistence of migraine symptoms such as photophobia and phonophobia [63], during and/or after COVID-19 was associated with an increased risk of long-term AIWS symptoms. Conversely, patients who experienced persistent headache with features indicative of cluster headache, such as autonomic symptoms like facial or forehead sweating and nasal congestion [63], during their acute COVID-19, were less likely to exhibit AIWS symptoms after COVID-19. The differential pathological mechanisms between migraines and cluster headache may explain the less frequent association and progression to AIWS observed with the latter [64, 65]. In the case of photophobia, thalamic trigeminovascular neurons play a direct role in receiving light impulses from the retina and transmitting them to cortical pain regions [65].

Brain functional magnetic resonance imaging analysis has revealed alterations in the temporo-occipital regions in patients with migraines and AIWS [66]. Brain positron emission tomography tracer 18 F-fluorodeoxyglucose studies have shown that patients with long COVID exhibit metabolic abnormalities in the temporal (amygdala, hippocampus, thalamus) and orbitofrontal regions [67, 68]. Similar abnormalities in the temporal and parieto-occipital regions have been observed in patients with depression and AIWS [69]. These temporal impairments align with visual, auditory, and memory alterations, which clinically correlate with overlapping symptoms across these pathologies. Individuals who exhibited depressive symptoms during their acute COVID-19 were also more likely to develop or maintain AIWS symptoms after COVID-19, providing further clinical evidence of a potential organic association and predisposition between these conditions. Moreover, major depressive disorder has been linked to a longer duration of AIWS symptoms [70], which aligns with our findings. Given the common metabolic abnormalities and associations observed in these pathologies across various brain regions, further neurological studies are warranted to identify the affected neurological pathways and explore potential therapeutic approaches.

Our findings carry significant clinical implications for the management of patients in the post-COVID-19 setting. The nearly tenfold increased odds of post-acute AIWS in patients who experienced these symptoms acutely suggests that the initial phase of SARS-CoV-2 infection is a critical window for the development of these perceptual disturbances. Clinicians should therefore consider asking patients about symptoms of perceptual distortion during acute COVID-19, as their presence may identify a cohort at high risk for long-term neuropsychiatric sequelae. Furthermore, the association of post-acute AIWS with symptoms like phonophobia, photophobia, and anxiety underscores the overlap with migrainous and psychiatric phenotypes. This suggests that post-COVID-19 care pathways should adopt a multidisciplinary approach, integrating neurology, psychiatry, and primary care [71]. Screening for these associated symptoms in patients presenting with persistent headache could serve as a practical tool to prompt further investigation for AIWS. Early identification would not only validate a patient’s often bewildering experiences but also facilitate targeted management strategies, which could include therapies established for migraine and anxiety disorders, potentially improving patient quality of life and reducing healthcare burden [72, 73].

From a public health perspective, our findings advocate for the formal recognition of AIWS as a potential component of post-acute sequelae of COVID-19 (PASC). Clinical guidelines for PASC management, which are still evolving, should incorporate modules on identifying and managing perceptual and dissociative disorders [23, 74]. Public health strategies could include the development and dissemination of educational materials for both healthcare providers and the public to raise awareness of AIWS, thereby reducing patient stigmatization and diagnostic delays. Furthermore, health systems could consider establishing specialized PASC clinics with integrated access to neurological and psychological services, a model that has been proposed to effectively manage the complex, multi-system nature of long COVID [71]. Our data, particularly the association with pre-existing migraine and depressive symptoms, may help inform risk stratification within these clinics, allowing for more efficient allocation of specialized resources to patients most likely to benefit. Documenting the prevalence and impact of syndromes like AIWS is a critical first step toward advocating for their inclusion in health policies and insurance coverage frameworks for long COVID rehabilitation.

This study has several important limitations that must be considered when interpreting the findings. First, the recruitment methodology, relying on online distribution via social media and institutional networks, is susceptible to selection bias. This approach may have preferentially attracted individuals who are more engaged with health-related online communities, are more technologically proficient, or are experiencing more severe or unusual symptoms, potentially leading to an overestimation of AIWS prevalence compared to the general post-COVID-19 population. The high proportion of healthcare workers (30.1%) in our sample may also reflect this bias and could influence reporting patterns due to greater health literacy or symptom awareness. However, our sensitivity analysis excluding this subgroup did not substantially alter the main findings. Second, our reliance on self-reported data introduces the possibility of recall bias, as participants were asked to recollect symptoms from their acute disease, and the accuracy of these memories may vary. Furthermore, headache type, including pre-existing migraine, and AIWS symptoms were based on participant self-report against provided definitions, not on formal clinical diagnosis by a healthcare professional using established criteria such as the International Classification of Headache Disorders, 3rd edition or the use of validated screening instruments such as the ID-Migraine. This limits the diagnostic precision of our headache classifications. Third, the data are cross-sectional and based on a survey instrument that, while pilot-tested for clarity, was not a formally validated diagnostic tool. This design does not allow for the establishment of causality. A critical limitation, therefore, is the inability to clinically differentiate the reported AIWS-like phenomena from other neurological mimics, such as persistent migraine aura, non-convulsive epileptic phenomena, or other functional disorders. The lack of clinical interviews, neurological examinations, and ancillary testing (e.g., EEG, neuroimaging) means our case identification is presumptive and the risk of misclassification cannot be quantified. This cross-sectional design prevents us from distinguishing whether these phenomena represent a form of migraine aura, postdromal effects, or an independent comorbidity. Fourth, the survey was targeted at individuals with persistent headache, meaning our findings on AIWS prevalence and its associated factors are specific to this population and cannot be generalized to all individuals recovering from COVID-19. Fifth, our use of a backward stepwise variable selection procedure is exploratory in nature. While this method is useful for identifying a parsimonious set of potential predictors from a large number of candidates, the *p* values of the final model should be interpreted with caution as the selection process itself may inflate the Type I error rate, and they do not possess the same properties as *p* values from a single, pre-specified model. Sixth, a further consideration is the external validity of our findings. Although our cohort was recruited from eight Latin American countries, the majority of respondents (69.9%) were from Venezuela. This demographic skew may limit the generalizability of our findings to other Latin American populations, which may differ in terms of healthcare access, genetic background, and circulating SARS-CoV-2 variants during the study period. While a formal subgroup analysis was not powered to detect significant differences, exploratory comparisons did not reveal major disparities in the prevalence of AIWS or its key predictors between Venezuelan and non-Venezuelan participants. Nevertheless, future multi-national studies with more balanced representation are needed to confirm these associations across the diverse populations of the region. Finally, as this study is cross-sectional, we lack longitudinal data on the evolution of these symptoms over time. Nevertheless, the study’s strengths encompass the wide and diverse study population included, the systematic way of data collection that prevented missing data or participants not meeting the inclusion criteria from being included, and the privacy that this method of data collection offered patients to avoid fear of stigmatization due to their symptoms. We believe the findings may provide useful information that could serve as the background for future research as an aid in the diagnosis of AIWS.

## Conclusions

This study makes three key contributions to the understanding of post-COVID-19 neurological syndromes. First, it provides the largest systematic characterization to date of AIWS in a cohort of patients with persistent headache after COVID-19, identifying common symptoms such as time distortion and derealization. Second, it establishes a strong statistical association between AIWS symptoms during acute infection and their persistence in the post-acute phase, identifying SARS-CoV-2 as a significant potential trigger for this syndrome. Third, it identifies a clinical phenotype at higher risk for post-COVID-19 AIWS—notably younger individuals with a history of migraine—and highlights associated symptoms like phonophobia and depression that may aid in clinical identification. While these findings are exploratory and require confirmation in prospective clinical studies, they underscore the importance of recognizing complex perceptual disorders within the spectrum of long COVID and provide a foundation for future research into their pathophysiology and management.

## Supplementary Information

Supplementary Material 1.

Supplementary Material 2.

Supplementary Material 3.

## Data Availability

All data generated or analyzed during this study are included in this published article and its supplementary information files.

## Abbreviations

AIWS: Alice in Wonderland syndrome
COVID-19: coronavirus disease 2019
SARS-CoV-2: severe acute respiratory syndrome coronavirus 2
SD: standard deviation
IQR: interquartile range
PASC: post-acute sequelae of COVID-19
