# Supplementary material for: Exploring Alice in Wonderland syndrome in adults with persistent headache after COVID-19: a cross-sectional study in Latin America
Source: BMC Neurol. 2025 Oct 2;25:408. doi: 10.1186/s12883-025-04422-y (PMC12492887; doi:10.1186/s12883-025-04422-y)
Supplement: Supplementary file 1 — Supplementary Material 1. [file 12883_2025_4422_MOESM1_ESM.docx]

**Supplementary Data 1.** AIWS symptom after SARS-CoV-2 infection by sex

| **Demographics** | **All (*n* = 106; 100%)** | **Female (*n* = 89; 84%)** | **Male (*n* = 17; 16%)** | ***p* value** |
| --- | --- | --- | --- | --- |
| Concomitant AIWS symptoms during acute COVID-19, yes (%) |  |  |  |  |
| Macrosomatognosia or microsomatognosia total or partial body | 18 (17) | 15 (16.9) | 3 (17.6) | 0.936^a^ |
| Macropsia or micropsia | 10 (9.4) | 7 (7.9) | 3 (17.6) | 0.206^a^ |
| Teleopsia or pelopsia | 19 (17.9) | 14 (15.7) | 5 (29.4) | 0.178^a^ |
| Derealization or depersonalization | 26 (24.5) | 25 (28.1) | 1 (5.9) | 0.051^a^ |
| Achromatopsia or hypochromatopsia | 4 (3.8) | 3 (3.4) | 1 (5.9) | 0.508^b^ |
| Time distortion | 34 (32.1) | 32 (36) | 2 (11.8) | 0.05^a^ |
| Dysmorphopsia | 10 (9.4) | 9 (10.1) | 1 (5.9) | 0.585^a^ |
| Hyperchromatopsia | 22 (20.8) | 19 (21.3) | 3 (17.6) | 0.73^a^ |
| Illusory levitation | 19 (17.9) | 15 (16.9) | 4 (23.5) | 0.511^a^ |
| Erythropsia | 6 (5.7) | 5 (5.6) | 1 (5.9) | 0.966^a^ |

^a^Pearson’s chi-square test; ^b^Fisher’s exact test.
