# Supplementary material for: Exploring Alice in Wonderland syndrome in adults with persistent headache after COVID-19: a cross-sectional study in Latin America
Source: BMC Neurol. 2025 Oct 2;25:408. doi: 10.1186/s12883-025-04422-y (PMC12492887; doi:10.1186/s12883-025-04422-y)
Supplement: Supplementary file 2 — Supplementary Material 2. [file 12883_2025_4422_MOESM2_ESM.docx]

**Supplementary Data 2.** Demographics of participants stratified by presence or absence of AIWS symptoms after COVID-19

| **Demographics** | **AIWS (*n* = 106; 25.2%)** | **Non-AIWS (*n* = 315; 74.8%)** | ***p* value** |
| --- | --- | --- | --- |
| Age, median (IQR), years | 36 (16) | 39 (19) | 0.011^a^ |
| Sex, *n* (%) |  |  | 0.446^b^ |
| Female | 89 (84) | 254 (80.6) |  |
| Male | 17 (16) | 61 (19.4) |  |
| Education level, *n* (%) |  |  | 0.273^c^ |
| None | 0 (0) | 1 (0.3) |  |
| Primary school | 0 (0) | 3 (1) |  |
| High school | 16 (15.1) | 28 (8.9) |  |
| Associate degree | 15 (14.2) | 37 (11.7) |  |
| University | 75 (70.8) | 246 (78.1) |  |
| Marital status, *n* (%) |  |  | 0.235^b^ |
| Single | 47 (44.3) | 120 (38.1) |  |
| Married | 42 (39.6) | 122 (38.7) |  |
| Common-law marriage | 14 (13.2) | 45 (14.3) |  |
| Divorced | 3 (2.8) | 18 (5.7) |  |
| Widowed | 0 (0) | 10 (3.2) |  |
| Race, *n* (%) |  |  | 0.279^c^ |
| Mixed | 61 (57.5) | 151 (47.9) |  |
| White | 44 (41.5) | 152 (48.3) |  |
| Black | 1 (0.9) | 8 (2.5) |  |
| Indigenous | 0 (0) | 4 (1.3) |  |
| Occupation, *n* (%) |  |  | 0.061^b^ |
| Healthcare worker | 32 (30.2) | 105 (33.3) |  |
| Employee | 29 (27.4) | 111 (35.2) |  |
| Self-employed | 25 (23.6) | 49 (15.6) |  |
| Student | 15 (14.2) | 25 (7.9) |  |
| Unemployed/Retired | 5 (4.7) | 25 (7.9) |  |
| Country of residence, *n* (%) |  |  | 0.961^c^ |
| Argentina | 2 (1.9) | 6 (1.9) |  |
| Chile | 1 (0.9) | 2 (0.6) |  |
| Colombia | 15 (14.2) | 34 (10.8) |  |
| Cuba | 0 (0) | 3 (1) |  |
| Ecuador | 1 (0.9) | 3 (1) |  |
| El Salvador | 0 (0) | 1 (0.3) |  |
| Mexico | 9 (8.5) | 39 (12.4) |  |
| Peru | 3 (2.8) | 7 (2.2) |  |
| Uruguay | 1 (0.9) | 4 (1.3) |  |
| Venezuela | 74 (69.9) | 215 (68.3) |  |

^a^Mann-Whitney U test; ^b^Pearson’s chi-square test; ^c^Fisher’s exact test.
