# Supplementary material for: Exploring Alice in Wonderland syndrome in adults with persistent headache after COVID-19: a cross-sectional study in Latin America
Source: BMC Neurol. 2025 Oct 2;25:408. doi: 10.1186/s12883-025-04422-y (PMC12492887; doi:10.1186/s12883-025-04422-y)
Supplement: Supplementary file 3 — Supplementary Material 3. [file 12883_2025_4422_MOESM3_ESM.docx]

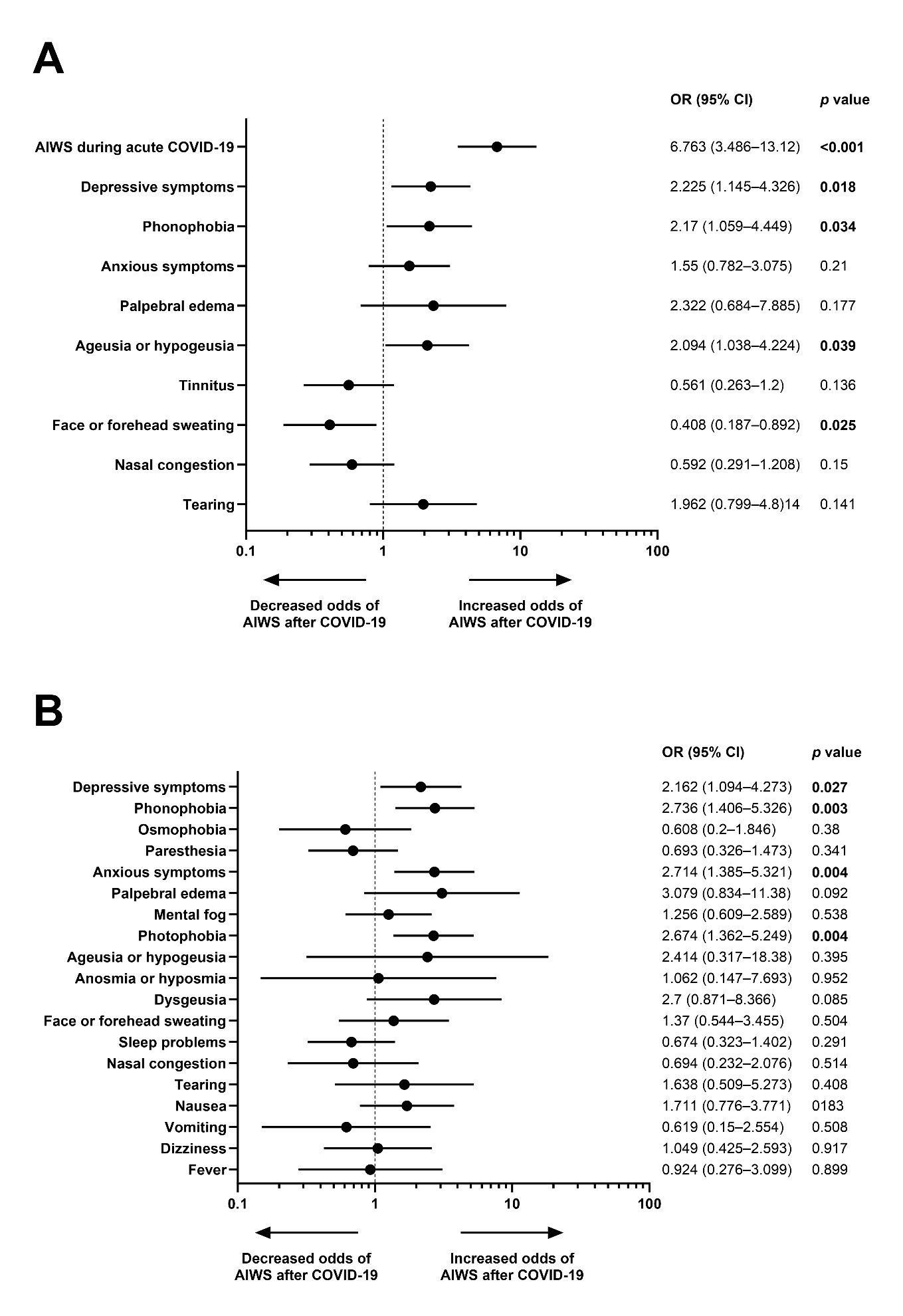


**Supplementary Data 3. Sensitivity analysis of factors associated with AIWS after COVID-19, excluding healthcare workers.** **A** Association between symptoms during acute COVID-19 and the odds of post-COVID-19 AIWS (*p* <0.001, R^2^ Nagelkerke = 0.335, Hosmer-Lemeshow test = 0.531, correctly classified 80.3% of the participants [338 out of 421]). **B** Association between concurrent post-COVID-19 symptoms and the odds of post-COVID-19 AIWS (*p* <0.001, R^2^ Nagelkerke = 0.33, Hosmer-Lemeshow test = 0.361, correctly classified 80.3% of the participants [338 out of 421]). Odds ratios (OR) > 1 indicate increased odds that a patient experienced post-COVID AIWS, while odds ratios < 1 indicate decreased odds. For each factor, the black circles represent the adjusted OR from the main analysis including all participants (*n* = 421), while the white squares represent the adjusted OR from the sensitivity analysis which excluded healthcare workers (*n* = 294). The lines represent the 95% confidence intervals for each estimate. For all symptoms listed, the reference category was the absence of that symptom. Variables with statistically significant associations (*p* <0.05) in the main analysis are indicated in bold
